# LT-FS-ID: Log-Transformed Feature Learning and Feature-Scaling-Based Machine Learning Algorithms to Predict the *k*-Barriers for Intrusion Detection Using Wireless Sensor Network

**DOI:** 10.3390/s22031070

**Published:** 2022-01-29

**Authors:** Abhilash Singh, J. Amutha, Jaiprakash Nagar, Sandeep Sharma, Cheng-Chi Lee

**Affiliations:** 1 Fluvial Geomorphology and Remote Sensing Laboratory, Indian Institute of Science Education and Research Bhopal, Bhopal 462066, India; sabhilash@iiserb.ac.in or; 2 Department of Electronics and Communication Engineering, School of ICT, Gautam Buddha University, Greater Noida 201312, India; roniamutha@gmail.com; 3 Subir Chowdhury School of Quality and Reliability, Indian Institute of Technology, Kharagpur 721302, India; jpnagar91@gmail.com; 4 Department of Electronics Engineering, Madhav Institute of Technology and Science, Gwalior 474005, India; 5 Department of Library and Information Science, Research and Development Center for Physical Education, Health, and Information Technology, Fu Jen Catholic University, New Taipei 242, Taiwan; 6 Department of Computer Science and Information Engineering, Asia University, Taichung 41354, Taiwan

**Keywords:** WSNs, intrusion detection, machine learning, feature learning, support vector regression

## Abstract

The dramatic increase in the computational facilities integrated with the explainable machine learning algorithms allows us to do fast intrusion detection and prevention at border areas using Wireless Sensor Networks (WSNs). This study proposed a novel approach to accurately predict the number of barriers required for fast intrusion detection and prevention. To do so, we extracted four features through Monte Carlo simulation: area of the Region of Interest (RoI), sensing range of the sensors, transmission range of the sensor, and the number of sensors. We evaluated feature importance and feature sensitivity to measure the relevancy and riskiness of the selected features. We applied log transformation and feature scaling on the feature set and trained the tuned Support Vector Regression (SVR) model (i.e., LT-FS-SVR model). We found that the model accurately predicts the number of barriers with a correlation coefficient (R) = 0.98, Root Mean Square Error (RMSE) = 6.47, and bias = 12.35. For a fair evaluation, we compared the performance of the proposed approach with the benchmark algorithms, namely, Gaussian Process Regression (GPR), Generalised Regression Neural Network (GRNN), Artificial Neural Network (ANN), and Random Forest (RF). We found that the proposed model outperforms all the benchmark algorithms.

## 1. Introduction

These days, security is one of the primary concerns for every nation caused by highly unpredictable and noxious events taking place across the globe [1,2,3]. Every nation wants to secure and protect its borders from any kind of intrusion and attack by enemy forces. In addition, unauthorised and illegal entry is another vital matter that requires immediate attention from the concerned authorities [4]. In order to protect their international borders from enemies and unfriendly forces, several nations have their regular armies. These army soldiers patrol along the border stretches, but patrolling methods are conventional, periodic, and limited. Since a country may have international boundaries that are thousands of miles long, it is impossible to deploy soldiers at every single location. Consequently, there remains a large area along the international borders that is unguarded. Enemies may take advantage of these unguarded locations and enter the territories. They can likely steal some classified documents crucial to the security of a nation, decimate defence personnel, or demolish crucial infrastructures. The surveillance and monitoring along the international borders and checkpoints can be achieved with the help of WSNs.

**Figure 1:**
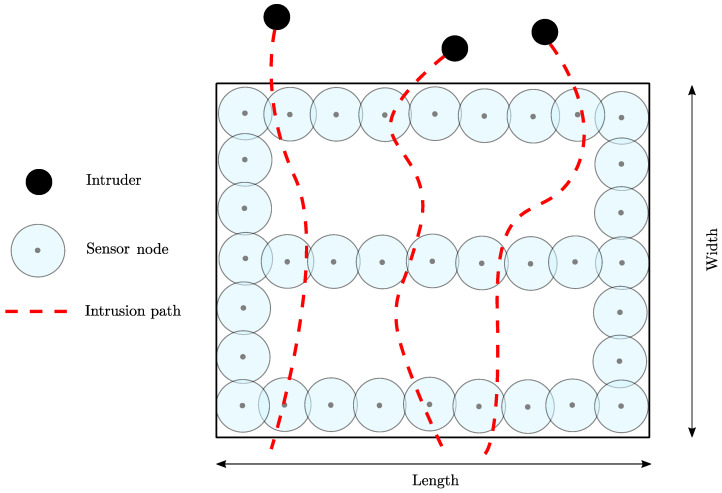
Illustration of 3-barrier coverage for each intrusion path.

WSNs is a widely accepted and renowned technology because it is cheap, readily available, and can be installed on the fly in almost no time at any place [5,6]. In addition, WSNs consist of small and homogeneous sensors that work in a de-centralised fashion requiring no pre-installed foundation and communicating over wireless channels [7]. Therefore, WSNs are employed for many civilian and military applications such as precision agriculture, health monitoring, structural health monitoring, industrial monitoring, disaster management, rescue operations, wild animal monitoring, landslide monitoring, fire detection, monitoring and surveillance in border areas, and many more [8,9,10,11]. Furthermore, intrusion detection in border areas and unauthorised access detection in restricted areas and infrastructures is a pivotal application of WSNs. For example, a WSN can be deployed to form a sensor barrier for any possible intrusion path as shown in Figure 1. The studies conducted so far on intrusion detection issues can be divided into two categories; first, it is described as a monitoring or surveillance system to detect an invader or an unauthorised entry in the RoI. Secondly, it is assumed to be a component of a WSN system specifically designed and implemented to diagnose compromised and/or vulnerable sensors for avoiding false alarms and ensuring correct network behaviour [12]. In this work, we concentrate on the first category.

The work presented in [13] proposed a fusion algorithm with three levels of hierarchy to spot a passive mobile intruder. They have employed two crucial modalities, namely the sensing probability model and acoustic signal model, to ascertain the presence of an invader. In addition, the authors have also analysed the influence of the number of sensors, intruder speed on the probability of detection, detection accuracy, and false alarm rate and found that the proposed algorithm outperforms the other fusion algorithms. Another work presented in [14] proposed optimal trajectories for mobile sensors employed for intrusion detection in a given RoI. The proposed trajectories for mobile sensors will maximise the coverage area and reduce energy consumption, which would increase the lifetime of the sensor network, thus providing improved intrusion detection performance. A distributed border surveillance system is proposed in [15], where the performance of the system is estimated in terms of the number of barriers obtained for a possible intrusion path in shadowed and non-shadowed environmental conditions. The authors found that the number of barriers obtained for shadowed environmental conditions is greater compared with the non-shadowed environmental conditions. Similarly, the work in [16] proposed a smart border surveillance system that uses ultrasonic, passive infra-red, and camera sensors to detect the presence of an intruder. The proposed system is capable of distinguishing between animal and human beings. The system sends an alert message and video streams to the control system as soon as it identifies an intruder. In Ref. [17], the authors have proposed a border surveillance system architecture that renders high energy efficiency and load balancing capabilities, thus, increasing the network lifetime. Furthermore, the proposed methodology needs less maintenance, involves low-cost installation, and delivers enhanced reliability. The authors claim that the proposed system outperforms other available intrusion detection systems and has an enhanced network lifetime. Another work provided in [18] presented an analytical model to detect a mobile intruder using mobile sensor networks. They have obtained an analytical formula to calculate the *k*-barrier coverage probability for an invader trying to cross a rectangular-belt region following a given path. They have also investigated the effect of network parameters such as sensor-to-intruder velocity ratio, sensing range, sensor count, and intrusion path angle on the performance metric. The proposed model is very effective in detecting an intrusion and tracking the enemy movements. Most recently, the authors in [19] proposed a remote surveillance system using robots with CCTV cameras. The authors claim that the proposed work will be useful for border surveillance and internal monitoring.

It is pivotal to mention that the above-discussed works [13,14,15,16,17,18,19] contribute significantly in the research domain. However, their models are validated through Monte Carlo simulation, which requires very high computation cost and time. For instance, it requires approximately 15 hours to achieve a single outcome through simulation runs at a given value of parameters. In addition, the simulation time increases exponentially with the increase in the number of sensors, sensing range and other network parameters. This is because of the fact that WSNs produce a large volume of data that requires plenty of time for its processing and analysis. Applications like infiltration in border regions are time-sensitive because a delay in seconds may cause catastrophes. Thus, it is vital to detect any kind of intrusion along the borders and around the prohibited regions as quickly as possible.

The problem at hand can be resolved by employing machine learning approaches that are exceptionally competent for computational time [20,21]. For instance, the work presented in [22] provided a mathematical framework to evaluate the *k*-barrier coverage probability for a given intrusion path using mobile WSNs. The authors have proposed three machine learning models based on the GPR algorithm to predict the *k*-barrier coverage probability to overcome the computational and time complexity problem. In doing so, they have considered sensing range, the number of sensors, sensor to intruder velocity ratio, mobile to static sensor ratio, required value of *k*, and intrusion path angle as potential features. The proposed machine learning model can predict the *k*-barrier coverage probability with higher accuracy than the other benchmark algorithms.

In this study, we proposed an efficacious machine learning-based approach to accurately predict the number of barriers for fast intrusion detection and prevention using relevant features. We extracted relevant features (i.e., the area of the RoI, sensing and transmission range of the sensor, and the total number of sensors) synthetically through Monte Carlo simulations. Subsequently, we applied feature transformation and scaling operations and trained a SVR model. We access the performance of the trained model by using R, RMSE, bias, and computational time complexity as the performance metrics. The main contributions of this paper are as follows:

- We introduced a synthetic data generation framework for a cost-effective solution.
- We estimated the relative importance score of each feature by using the regression tree ensemble approach.
- We performed the sensitivity analysis of the features using Partial Dependency Plot (PDP) analysis.
- We proposed a novel algorithm based on log-transformed feature learning and feature-scaling to accurately predict the number of barriers for fast intrusion detection and prevention. We also performed a sensitivity analysis of the proposed algorithm.

## 2. Material and Methods

### 2.1. Preparation of the Datasets

The performance of any machine learning model depends on the quality of datasets on which it is trained [23]. These datasets can either be field derived (obtained by direct measurements) or generated synthetically (obtained through simple rules, statistical modelling, and simulations) [24]. The use of synthetic data is increasing exponentially in the domain of healthcare [25,26], WSNs [22,27], and data privacy [28].

In this study, we extracted the datasets synthetically through simulations. To do so, we consider a finite number of sensors (N), distributed uniformly and randomly in a rectangular RoI. Each sensor is assumed to be homogeneous, i.e., sensing, transmission, and computational capabilities are identical for each sensor. The dimensions of the network deployment RoI are varied from 100 × 50 m^2^ to 250 × 200 m^2^. The entire dataset used for training and testing purposes is obtained through simulations using network simulator NS-2.35. The complete procedure for simulation outcomes is explained below.

Any two arbitrary sensors in the deployed WSN can communicate with each other, if they satisfy the condition, *R_tx_ ≥ 2R_s_*, where, *R_tx_* and *R_s_* indicates the transmission and sensing range of sensors respectively. Here, we have considered the most widely employed sensing range model known as the Binary Sensing Model (BSM) to estimate the performance of WSNs. According to BSM [29], a random sensor can detect a target with probability equal to one, if the target falls within the sensing range *R_s_* of the sensor denoted by *S_i_*. Otherwise, the target detection probability will be equal to zero. Mathematically, it can be represented by Equation (1).

(1) $$P_{det}=\left\{\begin{array}{c} 1,\;if\;d\left(S_{i},P\right)\leq R_{s}\\ 0,\;otherwise \end{array}\right.$$

where $d(S_{i},P)$ represents the Euclidean distance between the sensor $S_{i}$ and target point *P*. To identify the existence of intruders, a barrier is formed by connecting a sensor cluster over the entire RoI. To detect an intruder successfully, there should be at least one barrier for each possible intrusion path to ensure barrier coverage. The total number of sensors required to achieve the desired *k*-barrier coverage can be computed by *k* =$\left\lceil L/2R_{s}\right\rceil$ [1] and the maximum Barrier Paths (BP_max_) that can be constructed for a given intrusion path is computed as: BP_max_ = $\left\lfloor N/k\right\rfloor$, where *L* indicates the length of the rectangular RoI. The *k*-coverage ensures that each point in the target RoI is monitored by *k* distinct sensors, where *k* is a positive integer having value greater than one. Table 1 shows different network parameters and their values used to get the simulation results.

### 2.2. Calculation of Feature Importance and Sensitivity

To calculate each feature’s relative importance score, we created a regression ensemble through boosting ensemble learning. We leverage LSBoost (Least Square gradient Boosting) algorithm to boost hundred regression trees, each having unity learning rate [22,30]. This algorithm assumes each decision tree as a weak learner and processes them individually by identifying their weak points. Afterward, the algorithm process the next weak learner by concentrating on the weak aspect of the previous learner. In this way, the algorithm iteratively formed an ensemble of weak learners. Once the ensemble is generated, we calculated the feature importance by summing the total change in the normalised node risk.

Further, we performed the Partial Dependency Plot (PDP) analysis to assess the impact of each individual feature on the predictand. It computes the partial dependency of the considered feature set on the predictand by marginalising the impact of remaining features [27,30]. We considered a set of two features and computed their partial dependency on the predictand. For a set of four features, we have a total of six pairs of features. We plotted the 2D and 3D variation profiles.

### 2.3. SVR Model Set-Up

**Figure 2:**
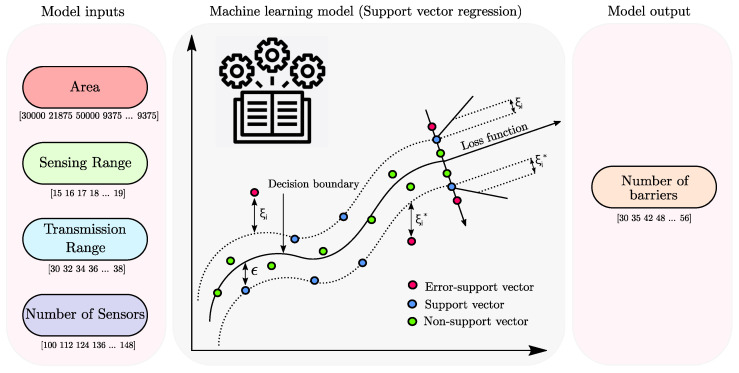
Illustration of the support vector regression with all input features and the corresponding response variable.

In this section, we have discussed the modelling of SVR [31,32] for the prediction of the number of barriers (Figure 2). It is an effective algorithm to address prediction problems, solve sample issues, and provide significant generalisation performance [30,33]. Using a nonlinear mapping φ (.): *ℜ*^*n*^→*ℜ*^*n*_*h*_^, the training sets (*x_i_, y_i_*), where *i* = 1 to *n*, are mapped into a high dimensional feature space, *ℜ*^*n*_*h*_^. Then, a linear function, *f*, is used to express the nonlinear association among features and the response variable. The SVR function [34] is a linear function which is represented as:

(2) $$f\left(x\right)=w^{T}\varphi \left(x\right)+B$$

where *f(x)* indicates the forecasting values, *w ∈ℜ*^*n*_*h*_^ indicates the weighting matrix, and *B ∈ℜ* indicates the bias term. The SVR approach intends to reduce the empirical risk as:

(3) $$R_{em}\left(f\right)=\frac{1}{N}\sum_{i=1}^{N}\Theta_{\epsilon }\left(y_{i},w^{T}\varphi \left(x_{i}\right)+B\right)$$

where Θ_ϵ_(*y*_*i*_, *w*^*T*^φ(*x*_*i*_) + *B*) indicates the ϵ-insensitive loss function that determines the optimal hyper plane on a high-dimensional feature space to maximise the distance between two subsets of input dataset. It is determined by:

(4) $$\Theta_{\epsilon }\left(y_{i},w^{T}\varphi \left(x_{i}\right)+B\right)=\left\{\begin{array}{c} w^{T}\varphi \left(x_{i}\right)+B-y_{i}-\epsilon \;if\;w^{T}\varphi \left(x_{i}\right)+B-y_{i}\geq \epsilon \\ 0,\;otherwise \end{array}\right.$$

Hence, SVR is concerned with identifying the optimal hyper plane and decreasing the residual between the training datasets and the ϵ-insensitive loss function. Moreover, SVR reduces the total errors by:

(5) $$\min_{w,B,\xi^{*},\xi }R_{\epsilon }\left(w,\xi^{*},\xi \right)=\frac{1}{2}w^{T}w+C\sum_{i=1}^{N}\left(\xi_{i}^{*}+\xi_{i}\right)$$

with the following constraints

$$\begin{array}{c} y_{i}-w^{T}\varphi \left(x_{i}\right)-B\leq \epsilon +\xi_{i}^{*},\;i=1,2,\ldots ,N\\ -y_{i}+w^{T}\varphi \left(x_{i}\right)+B\leq \epsilon +\xi^{i},\;i=1,2,\ldots ,N\\ \xi_{i}^{*}\geq 0,\;i=1,2,\ldots ,N\\ \xi_{i}\geq 0,\;i=1,2,\ldots ,N \end{array}$$

Equation (4) normalises weight sizes, ensures regression function flatness, penalises *f(x)* and *y* training residuals by the ϵ-insensitive loss function, and *C* represents the penalty parameter. Training residuals above ϵ are represented as $\xi_{i}^{*}$ and below −ϵ are represented as $\xi_{i}$. However, in the dual space, SVR function is represented as:

(6) $$f\left(x\right)=\sum_{i=1}^{N}\left(\beta_{i}^{*}-\beta_{i}\right)K\left(x_{i},x_{j}\right)+B$$

where *K(x_i_,x_j_)* represents the kernel function. It is the inner product of *x_i_* and *x_j_* vectors in the feature space φ(*x_i_)* and φ(*x_j_)*, respectively. We have used polynomial kernel (Equation (7)) as it belongs to the group of the non-stationary kernel that performs effectively over standarised and transformed features [35].

(7) $$K\left(x_{i},x_{j}\right)=\gamma {\left(\left(x_{i}\cdot x_{j}\right)+1\right)}^{\omega }$$

where γ and ω are the kernel function’s structural parameter and polynomial degree, respectively. The prediction accuracy of an SVR model is governed by the good tuning of hyperparameters (*C* and ϵ). If the residual between the observed and predicted value is greater than the hyperparameter ϵ then the other hyperparameter *C*, penalises the model. Hence, a high value of *C* results in under-fitting, and a lower value leads to high computational complexity [27].

In this study, we applied the universal grid optimisation algorithm [36] to optimise the hyperparameters. We selected the most frequently used Mean Square Error (MSE) function [37] as the objective function given by:

(8) $$\frac{1}{n}\sum_{i=1}^{n}{\left(f_{i}-\hat{f_{i}}\right)}^{2}$$

where *n* is the sampling size, $f_{i}$ is the observed and $\hat{f_{i}}$ is the predicted values. We iteratively optimised *C* for all possible ϵ by considering the MSE function as the objective function. We found the optimal value of *C* = 0.1 and ϵ = 0.01. Afterward, we applied log transformation (LT) [38] and mean z-score scaling (Equation (9)) on the input features. Where $x_{f}$ is the input feature set, $\bar{x_{f}}$ is the mean of the feature set, and σ is the standard deviation of the feature set.

(9) $$x_{sf}=\frac{x_{f}-\bar{x_{f}}}{\sigma }$$

**Figure 3:**
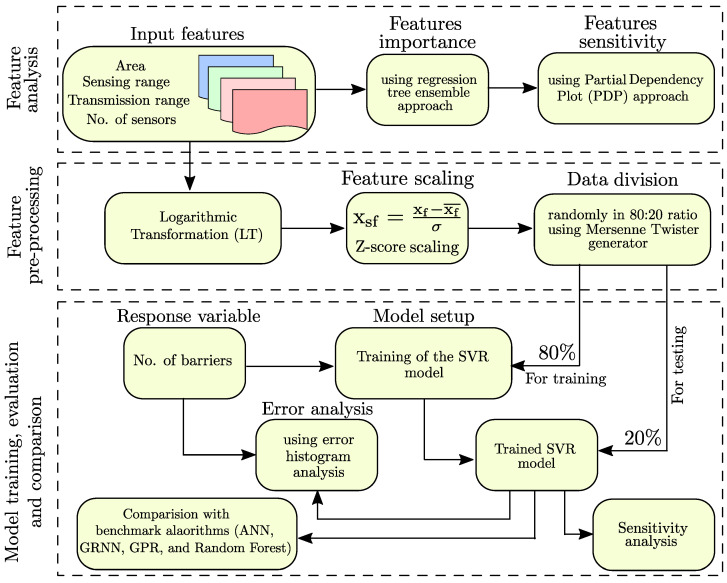
Flowchart of the proposed methodology.

Once we applied feature pre-processing, we trained and evaluated the SVR model in an 80:20 ratio. The datasets are divided randomly using Mersenne Twister random generator. We illustrated the complete methodology in Figure 3 and also enumerated the complete process into the following steps;

1. We synthetically generated the input features (i.e., area of the RoI, sensing range of the sensors, transmission range of the sensor, and the number of sensors) through Monte Carlo simulations.
2. We trained a regression tree ensemble to estimate each feature’s relative feature importance score.
3. We leverage PDP analysis to perform the sensitivity analysis of each feature.
4. We applied feature scaling on the selected features post log transformation.
5. We used the Mersenne Twister generator with a random seed to randomly divide the datasets for training and testing the model in a ratio of 80:20.
6. We used 80% of the datasets to set up the machine learning model.
7. We used the remaining 20% of the datasets to test the performance of the trained model.
8. We performed the sensitivity analysis of the trained model.
9. We performed the error analysis using error histogram analysis to understand the distribution of the errors.
10. We compared the performance of the trained model with the benchmark algorithms (i.e., ANN, GRNN, GPR, and Random Forest).

## 3. Results

In this section, firstly, we discuss the results of feature importance and sensitivity analysis. Afterward, we discuss the performance of the proposed model.

### 3.1. Feature Importance and Sensitivity

**Figure 4:**
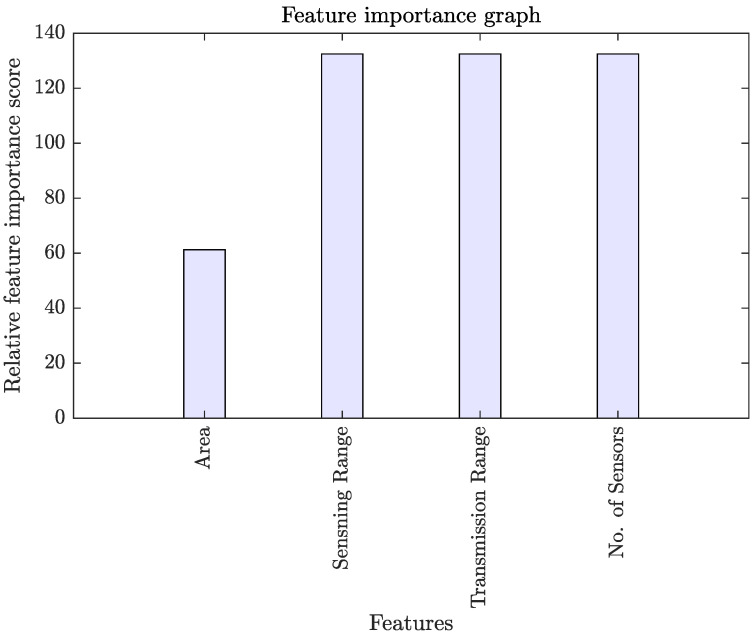
Bar graph illustrating each feature’s relative feature importance score estimated through regression tree ensemble approach.

We evaluated the prominence of each feature through the regression tree ensemble approach. The bars in Figure 4 show the relative feature importance score of each feature. The feature importance score of all four features ranges between 60 to 140. We found that the area of the RoI has the least feature importance among all, indicating that area of the rectangular region is the least relevant feature in predicting the number of barriers for fast intrusion detection and prevention. Surprisingly, we found that the sensing range of the sensor, the transmission range of the sensor, and the number of sensors have the same and highest feature importance score, indicating that they are the most relevant features in predicting the number of barriers.

**Figure 5:**
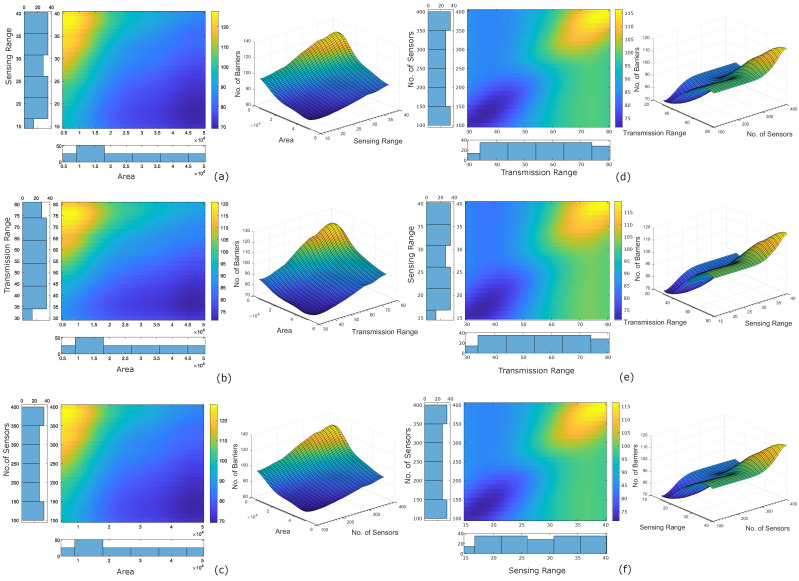
Feature sensitivity analysis through partial dependency plot. Two features are considered at a time (a total of six pairs from **a**–**f**). The left image shows the 2-D variation profile (with histogram) for each pair, and the right image shows the corresponding 3-D variation profile.

Further, we performed the feature sensitivity analysis of all the four features through the Partial Dependency Plot (PDP) analysis (Figure 5). We observed that area of the rectangular region has a negative repercussion on the response variable (i.e., number of barriers). In contrast, the sensing range of the sensors, the transmission range of the sensors, and the number of sensors have a positive repercussion on the response variable.

### 3.2. Model Performance

**Figure 6:**
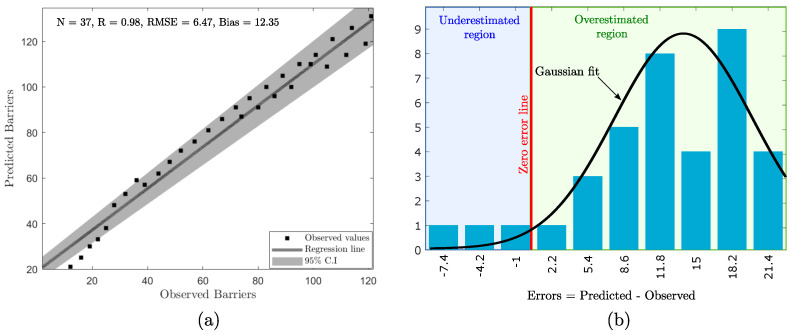
(**a**) Linear regression curve between the predicted result of LT-ZM-SVR model and observed values. (**b**) Error distribution analysis through error histogram.

Once our model is trained, we evaluate its performance by using R, RMSE, and bias as the performance metrics. To do so, we fed the testing datasets into the trained model’s input and obtained the predicted response from the model. Afterward, we plotted a linear fit line between the observed and predicted response variable in Figure 6a. In doing so, we observed that the predicted values accord well with the observed values (R = 0.98, RMSE = 6.47, and bias = 12.35). All the data points lie around the regression line, with very few (especially the lower values) lying beyond the 95% Confidence Interval (C.I.). However, the presence of positive bias indicates that the model is slightly overestimating the response variable.

Further to understand the distribution of errors in the model, we have plotted the error histogram of the model using 10 bins (Figure 6b). We fitted a continuous Gaussian fit on the error distribution and found that the error follows left-skewed distribution (also called negatively skewed distribution). The error ranges from −7.4 (leftmost bin) to 21.4 (rightmost bin). Negative errors (left to the zero error line) represent the underestimated region, and positive errors (right to the zero error line) represent the overestimated region. The peak of the distribution lies in the overestimated region, indicating the presence of positive bias.

## 4. Discussion

### 4.1. Comparison with Other Scaling Methods

We also evaluated and compared the performance of other scaling approaches. We considered Center Mean (CM) scaling and Min-Max scaling along with Z-score scaling. We also considered the Non-Scaled (NS) version for an appropriate comparison. After log transformation of the features, we applied these scaling techniques and trained the SVR model. We reported the performance of LT-NS-SVR, LT-CM-SVR, LT-ZM-SVR, and LT-MM-SVR in Table 2. Interestingly, we found that the predicted barriers accord well with the observed values for all the variants. However, the RMSE, MSE, bias, and computational time complexity of LT-NS-SVR is worst among all.

### 4.2. Comparison with Benchmark Algorithms

To ensure an unbiased conclusion, we compared the performance of the proposed approach with different benchmark algorithms. In doing so, we evaluated the performance of ANN [39], GRNN [40], GPR [41,42], and Random Forest (RF) [43] over the same datasets after performing LT and z-score scaling on the features (Table 3). These models are selected based upon their performance in different applications such as remote sensing [30], WSNs [44], IoT [45], and blockchain [46]. We selected R, RMSE, MSE, bias, and computational time as the performance metrics. In comparing, we found that the proposed approach outperforms the benchmark algorithms in terms of RMSE, MSE, and bias. Additionally, LT-ZM-SVR emerges as the computationally efficient approach. Surprisingly, we found that the RF has the best R; however, with a poor RMSE. We observed a positive bias (i.e., overestimation tendency) in GRNN, GPR, RF, and LT-ZM-SVR. In contrast, a negative bias (i.e., underestimation tendency) is observed with ANN.

### 4.3. Sensitivity Analysis of the LT-ZM-SVR

**Figure 7:**
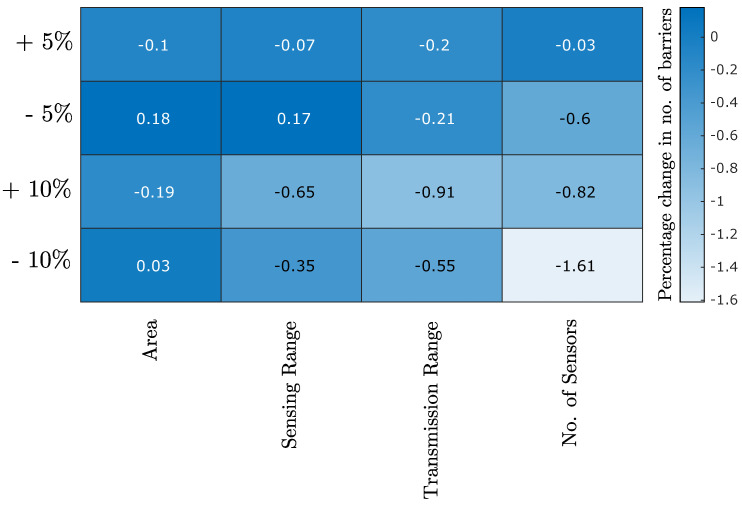
Sensitivity analysis of LT-ZM-SVR for ±5% and ±10% uncertainty in the input feature.

Finally, we performed the sensitivity analysis of the LT-ZM-SVR model to evaluate its robustness in the presence of uncertainty in input features. To do so, we introduced a fixed amount of variation in any one of the input features, keeping others constant. We performed this iteratively for all the features and reported the percentage change in the response variable in Figure 7. From the heat map, we found that overall the model is quite stable in the presence of small uncertainty. Relatively, the model is more vulnerable to the uncertainly present in the number of sensors.

## 5. Conclusions

This study proposed a novel approach to estimate the number of barriers required for intrusion detection. To do so, we extracted relevant features from the network parameters through Monte Carlo simulations. We evaluated the relevancy of each feature through feature importance analysis. We found the area of the RoI to be the least relevant feature in estimating the number of barriers. All other features (*i.e.*, the sensing range, the transmission range, and the number of sensors) equally carry the highest relevancy. Additionally, to measure the impact of each feature on the response variable, we performed a feature sensitivity analysis. We observed that except for the area of the RoI, all other features positively impact the response variable. Afterward, we applied log transformation and scaling operations on the selected features. After feature pre-processing, we applied the tuned SVR algorithm as an interpretable data-driven model. Once our model was trained, we evaluated its performance on the testing datasets using R, RMSE, MSE, bias, and computational time complexity as performance metrics. We found that the proposed approach accurately and timely predicts the number of barriers for fast intrusion detection and prevention.

For a robust conclusion, we compared the performance of the proposed approach with different scaling and benchmark algorithms. We found that the proposed methodology outperforms all the benchmark algorithms. However, the limitation of the proposed algorithm is that it assumes the values of the input features to be a positive real number. This study is a step towards fast intrusion detection and prevention using WSNs. Our approach can be employed for near-real-time applications such as border surveillance.

## Figures and Tables

**Table 1:** Simulation parameters.

Parameters	Values
Simulator	NS-2.35
Network region	Rectangular RoI
Network area ($m^{2}$)	100 × 50 to 250 × 200
Number of sensors (*N*)	100 to 400
Sensing range (*R_s_*)	15 to 40 m
Transmission range (*R_tx_*)	30 to 80 m
Sensor’s deployment type	Uniform distribution
Sensing model	Binary sensing model

**Table 2:** Comparison of the performance of Z-score scaling (i.e., LT-ZM-SVR) with other scaling methods (i.e., LT-NS-SVR, LT-CM-SVR, and LT-MM-SVR).

Performance Metrics	LT-NS-SVR	LT-CM-SVR	LT-ZM-SVR	LT-MM-SVR
R	0.96	0.94	0.98	0.97
RMSE	12.66	2.39	6.47	4.59
MSE	160.15	5.727	41.87	21.10
Bias	36.30	6.24	12.35	15.62
Time (s)	2.21	0.59	0.65	0.51

**Table 3:** Comparison of the proposed model with the benchmark algorithms.

Performance Metrics	Methods				
	LT-ZM-SVR	ANN	GRNN	GPR	Random Forest
R	0.98	0.38	0.96	0.94	0.99
RMSE	6.47	46.37	57.56	63.83	32.15
MSE	41.87	2150.20	3312.00	4074.7	1033.6
Bias	12.35	-36.12	49.62	50.96	28.62
Time (s)	0.65	1.81	2.02	1.71	2.70

## Data Availability

The datasets generated during and/or analysed during the current study can be downloaded from https://www.kaggle.com/abhilashdata/intrusion-data-wsn (accessed on 26 January 2022).

## References

[1] Mostafaei H., Chowdhury M.U., Obaidat M.S. (2018). Border surveillance with WSN systems in a distributed manner. IEEE Syst. J..

[2] Lee S., Jain S., Yuan Y., Zhang Y., Yang H., Liu J., Son Y.J. (2019). Design and development of a DDDAMS-based border surveillance system via UVs and hybrid simulations. Expert Syst. Appl..

[3] Sharma M.K., Singal G., Gupta S.K., Chandraneil B., Agarwal S., Garg D., Mukhopadhyay D. INTERVENOR: Intelligent Border Surveillance using Sensors and Drones. Proceedings of the 2021 6th International Conference for Convergence in Technology (I2CT).

[4] Komar C., Donmez M.Y., Ersoy C. (2012). Detection quality of border surveillance wireless sensor networks in the existence of trespassers’ favorite paths. Comput. Commun..

[5] Nagar J., Chaturvedi S.K., Soh S. (2021). An analytical framework with border effects to estimate the connectivity performance of finite multihop networks in shadowing environments. Cluster Comput..

[6] Singh A., Sharma S., Singh J., Kumar R. (2019). Mathematical modelling for reducing the sensing of redundant information in WSNs based on biologically inspired techniques. J. Intell. Fuzzy Syst..

[7] Nagar J., Chaturvedi S.K., Soh S. (2021). Wireless Multihop Network Coverage Incorporating Boundary and Shadowing Effects. IETE Tech. Rev..

[8] Singh A., Sharma S., Singh J. (2021). Nature-inspired algorithms for wireless sensor networks: A comprehensive survey. Comput. Sci. Rev..

[9] Kandris D., Nakas C., Vomvas D., Koulouras G. (2020). Applications of wireless sensor networks: An up-to-date survey. Appl. Syst. Innov..

[10] Kotiyal V., Singh A., Sharma S., Nagar J., Lee C.C. (2021). ECS-NL: An Enhanced Cuckoo Search Algorithm for Node Localisation in Wireless Sensor Networks. Sensors.

[11] Amutha J., Sharma S., Sharma S.K. (2021). Strategies based on various aspects of clustering in wireless sensor networks using classical, optimization and machine learning techniques: Review, taxonomy, research findings, challenges and future directions. Comput. Sci. Rev..

[12] Wang Y., Fu W., Agrawal D.P. (2012). Gaussian versus uniform distribution for intrusion detection in wireless sensor networks. IEEE Trans. Parallel Distrib. Syst..

[13] Sharma A., Chauhan S. (2020). Sensor fusion for distributed detection of mobile intruders in surveillance wireless sensor networks. IEEE Sens. J..

[14] Nurellari E., Licea D.B., Ghogho M., Rivero-Angeles M.E. (2021). On Trajectory Design for Intruder Detection in Wireless Mobile Sensor Networks. IEEE Trans. Signal Inf. Process. Netw..

[15] Amutha J., Nagar J., Sharma S. (2021). A distributed border surveillance (dbs) system for rectangular and circular region of interest with wireless sensor networks in shadowed environments. Wirel. Pers. Commun..

[16] Singh R., Singh S. (2021). Smart border surveillance system using wireless sensor networks. Int. J. Syst. Assur. Eng. Manag..

[17] Vadivelan N., Taware M.S., Chakravarthi M.R.R., Palagan C.A., Gupta S. (2021). A border surveillance system to sense terrorist outbreaks. Comput. Electr. Eng..

[18] Sharma S., Nagar J. (2020). Intrusion detection in mobile sensor networks: A case study for different intrusion paths. Wirel. Pers. Commun..

[19] Karthy G., Harish M., Harish R., Srivarshan R.N., Sridhar B. BORS (Border Patrol Search) ROBOT by using Wireless Technology. Proceedings of the 2021 Second International Conference on Electronics and Sustainable Communication Systems (ICESC).

[20] Roman R.C., Precup R.E., Petriu E.M. (2021). Hybrid data-driven fuzzy active disturbance rejection control for tower crane systems. Eur. J. Control.

[21] Zhu Z., Pan Y., Zhou Q., Lu C. (2020). Event-triggered adaptive fuzzy control for stochastic nonlinear systems with unmeasured states and unknown backlash-like hysteresis. IEEE Trans. Fuzzy Syst..

[22] Singh A., Nagar J., Sharma S., Kotiyal V. (2021). A Gaussian process regression approach to predict the k-barrier coverage probability for intrusion detection in wireless sensor networks. Expert Syst. Appl..

[23] Schmidt J., Marques M.R., Botti S., Marques M.A. (2019). Recent advances and applications of machine learning in solid-state materials science. Npj Comput. Mater..

[24] Nikolenko S.I. (2019). Synthetic data for deep learning. arXiv.

[25] Chen R.J., Lu M.Y., Chen T.Y., Williamson D.F., Mahmood F. (2021). Synthetic data in machine learning for medicine and healthcare. Nat. Biomed. Eng..

[26] Rankin D., Black M., Bond R., Wallace J., Mulvenna M., Epelde G. (2020). Reliability of supervised machine learning using synthetic data in health care: Model to preserve privacy for data sharing. JMIR Med. Inform..

[27] Singh A., Kotiyal V., Sharma S., Nagar J., Lee C.C. (2020). A machine learning approach to predict the average localization error with applications to wireless sensor networks. IEEE Access.

[28] Abay N.C., Zhou Y., Kantarcioglu M., Thuraisingham B., Sweeney L. Privacy preserving synthetic data release using deep learning. Proceedings of the Joint European Conference on Machine Learning and Knowledge Discovery in Databases.

[29] Zou Y., Chakrabarty K. (2004). Sensor deployment and target localization in distributed sensor networks. ACM Trans. Embed. Comput. Syst. (TECS).

[30] Singh A., Gaurav K., Rai A.K., Beg Z. (2021). Machine learning to estimate surface roughness from satellite images. Remote Sens..

[31] Vapnik V. (2013). The Nature of Statistical Learning Theory.

[32] Cortes C., Vapnik V. (1995). Support-vector networks. Mach. Learn..

[33] Garg R., Kumar A., Bansal N., Prateek M., Kumar S. (2021). Semantic segmentation of PolSAR image data using advanced deep learning model. Sci. Rep..

[34] Hong W.C. (2011). Electric load forecasting by seasonal recurrent SVR (support vector regression) with chaotic artificial bee colony algorithm. Energy.

[35] Heinonen M., Mannerström H., Rousu J., Kaski S., Lähdesmäki H. (2016). Non-stationary gaussian process regression with hamiltonian monte carlo. Artificial Intelligence and Statistics.

[36] Syarif I., Prugel-Bennett A., Wills G. (2016). SVM parameter optimization using grid search and genetic algorithm to improve classification performance. Telkomnika.

[37] Reed R., MarksII R.J. (1999). Neural Smithing: Supervised Learning in Feedforward Artificial Neural Networks.

[38] Zhan T., Gong M., Jiang X., Li S. (2018). Log-based transformation feature learning for change detection in heterogeneous images. IEEE Geosci. Remote Sens. Lett..

[39] Benardos P., Vosniakos G.C. (2007). Optimizing feedforward artificial neural network architecture. Eng. Appl. Artif. Intell..

[40] Specht D.F. (1991). A general regression neural network. IEEE Trans. Neural Netw..

[41] Rasmussen C.E. (2003). Gaussian Processes in Machine Learning. Advanced Lectures on Machine Learning.

[42] Quinonero-Candela J., Rasmussen C.E. (2005). A unifying view of sparse approximate Gaussian process regression. J. Mach. Learn. Res..

[43] Breiman L. (2001). Random forests. Mach. Learn..

[44] Zhang D., Zhang X., Xie F. (2021). Research on Location Algorithm Based on Beacon Filtering Combining DV-Hop and Multidimensional Support Vector Regression. Sensors.

[45] Gupta N., Khosravy M., Patel N., Dey N., Crespo R.G. (2021). Lightweight Computational Intelligence for IoT Health Monitoring of Off-Road Vehicles: Enhanced Selection Log-scaled Mutation GA Structured ANN. IEEE Trans. Ind. Inf..

[46] Dibaei M., Zheng X., Xia Y., Xu X., Jolfaei A., Bashir A.K., Tariq U., Yu D., Vasilakos A.V. (2021). Investigating the prospect of leveraging blockchain and machine learning to secure vehicular networks: A survey. IEEE Trans. Intell. Transp. Syst..

